# An Analysis of Acquisition Dose in Male Mammography

**DOI:** 10.1002/jmrs.70027

**Published:** 2025-10-15

**Authors:** Zechary Ng, Maeve Masterson, Daniel Carrion, Mohamed K. Badawy

**Affiliations:** ^1^ Department of Medical Radiations, School of Health and Biomedical Sciences RMIT University Bundoora Victoria Australia; ^2^ Monash Health Imaging Monash Health Melbourne Victoria Australia; ^3^ Department of Medical Imaging and Radiation Sciences, School of Primary and Allied Health Care, Faculty of Medicine, Nursing and Health Sciences Monash University Melbourne Victoria Australia

**Keywords:** DBT, ESAK, FFDM, gender‐specific protocols, glandular dose, male mammography, radiation dose

## Abstract

**Introduction:**

Although primarily used for female patients, mammography is a valuable tool for detecting male breast abnormalities, providing detailed images of breast tissue. Due to the low incidence of male breast imaging, limited male‐specific studies exist on radiation exposure. This study measured the acquisition radiation dose of male patients undergoing full‐field digital mammography (FFDM) and digital breast tomosynthesis (DBT) at an Australian tertiary hospital and compared these values with published cohorts. The goal was to determine whether differences in breast anatomy between sexes influenced radiation dose levels.

**Methods:**

A retrospective analysis of 266 male breast imaging acquisitions (66 patients, 78 procedures) across two hospital sites between February 2019 and April 2024 was performed. Both FFDM (*n* = 164) and DBT (*n* = 102) were assessed. Radiation dose metrics, including entrance surface air kerma (ESAK) and glandular dose (GD), were extracted from DICOM headers using a Python script. Data was anonymised and analysed with descriptive statistics, Shapiro–Wilk, Mann–Whitney *U*, and Pearson correlation to compare modalities and explore associations between patient and technical factors.

**Results:**

Median participant age was 65 years; median breast thickness 51 mm. Median FFDM variables: 28 kVp, 128 mAs, 570 ms exposure time, 2.7 mGy ESAK, 0.83 mGy GD. For DBT: 29 kVp, 100 mAs, 975 ms exposure time, 4.7 mGy ESAK, 1.34 mGy GD. Shapiro–Wilk (*p* < 0.001) confirmed non‐normal distributions. DBT doses were significantly higher than FFDM (Mann–Whitney *U*, *p* < 0.001). FFDM GD values were lower than both male and female cohorts reported previously. Breast thickness correlated strongly with FFDM ESAK (Pearson *r* = 0.72, *p* < 0.001).

**Conclusions:**

This study quantified ESAK and GD in male FFDM and DBT patients and compared these values with published female cohorts. Male doses were consistently lower, likely from reduced breast thickness and density. This study successfully measured acquisition dose in male mammography patients, identifying differences by sex and highlighting breast anatomy's influence.

## Graphical abstract

This retrospective study analysed 266 male breast imaging acquisitions across two Australian hospital sites, comparing radiation doses from full‐field digital mammography (FFDM) and digital breast tomosynthesis (DBT). DBT doses were significantly higher than FFDM, while FFDM glandular doses were lower than previously reported male and female cohorts. Findings suggest reduced breast thickness and density in men contribute to consistently lower radiation doses.

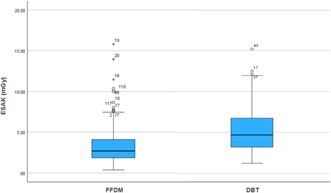

## 1 Introduction

Breast cancer is significantly less common in males than in females at a rate of approximately 1 in 100,000, and although rare, delayed diagnosis and increased advanced disease at diagnosis [1] underline the need for optimised imaging strategies in men. Full‐field digital mammography (FFDM), the primary technique for conventional breast imaging, typically involves a cranio‐caudal and medio‐lateral oblique acquisition of each breast. FFDM, however, incurs a higher number of false positive findings [2], produces poor images in patients with dense breast tissue [3] and as a 2D image, can face difficulty in accurately localising abnormalities [2]. Digital breast tomosynthesis (DBT), typically used diagnostically following FFDM, overcomes these challenges at the expense of increased radiation dose. DBT combines multiple acquisitions across an arc angle to create a 3D image of the breast with less noise and more detail than FFDM, even in patients with dense breast tissue [4]. The ability to view tissue without anatomical overlap improves nodule visibility and lesion detection [5], as highlighted in the Storm Trial [6], with a 5.3% increase in cancer detection rate and a 17% reduction in false positives compared to FFDM. These diagnostic benefits decrease the likelihood of needing additional imaging, resulting in a substantially lower recall rate for women attending consecutive screenings [7].

Current exposure settings for FFDM and DBT are optimised for the female breast, which differs from the average male breast that primarily consists of skin, fat, and fibrous tissues with fewer ducts [8]. Without further research, efficient radiation use in male breast imaging cannot be achieved. This highlights the importance of studying gender‐based differences in breast composition, particularly given the absence of agreed protocols for male mammography and the current risk of overexposing male breast tissue without corresponding clinical benefit [8]. As DBT continues to expand in clinical practice due to its superior diagnostic accuracy, evaluating its radiation dose implications for male patients is especially important.

Whilst there is no current research on this specific topic, Baek et al. [9] explored the need for unique radiation dose management for Korean women who typically had smaller, yet increased breast density, leading to an average increased average glandular dose (AGD) per examination relative to dose studies on Western women. Retrospective and prospective searching of literature has highlighted the lack of studies surrounding male‐specific radiation dose in mammography, with only Sulieman et al. [10] studying this. The study analysed radiation dose data of 42 male patients in Riyadh, Saudi Arabia, and concluded a comparable dose amount between men and women. It determined an average entrance surface dose of 5.3 mGy per acquisition, comparing it to the dose range of 5–8.98 mGy in previous female studies. However, this remains the only study to date addressing male‐specific mammography dose, and its limited sample size and lack of population diversity restrict generalisability. Furthermore, there is an absence of studies with Australian data and a scarcity of studies assessing male DBT dose. These knowledge gaps reinforce the need for further research in this area.

This study aims to quantify radiation doses in male patients undergoing FFDM and DBT in an Australian hospital setting, and to compare these results with previously reported female cohorts. By doing so, this study aims to investigate whether the sex of the breast anatomy contributes to dose levels.

## 2 Materials and Methods

This retrospective study examined male mammography dose data from procedures performed at two separate sites of a large tertiary hospital, referred to as Site 1 and Site 2. Non‐probability convenience sampling of health records was used, whereby all male breast imaging acquisitions performed at either site between January 2019 and April 2024 were screened for eligibility and included where criteria were met. Duplicate records, incomplete studies, and cases outside the inclusion period were excluded. Relevant cases were filtered, identified, and shortlisted from the picture archiving and communication system (PACS) using inclusion and exclusion criteria, shown in Table 1.

**TABLE 1:** Visualisation of the script's inclusion and exclusion criteria.

Inclusion criteria	Exclusion criteria
Examination years from 2019 to 2024	Biopsy procedures
Examination at tertiary centre, from Site 1 or 2	Cases with missing radiation dose information
2D Mammography procedures	
3D Tomosynthesis procedures	
Male	

Following filtering, the final data set used for analysis included 66 male patients across 78 procedures, including 56 FFDM and 22 DBT, resulting in 266 acquisitions. Site 1 contributed 102 acquisitions (15 FFDM and 87 DBT), while Site 2 accounted for 164 FFDM acquisitions and no DBT. Two acquisitions lacked available glandular dose data, leaving 264 analysable glandular dose acquisitions.

A Python script, developed using Python 3.10 and Pydicom 2.4, was created to retrieve data from DICOM headers according to the DICOM protocol standard. The script utilized the Pydicom module for data extraction, compiling key information and exporting it to a Microsoft Excel spreadsheet via the Pandas module 2.2.

The extracted data included patient identification numbers, radiation dose values, study and series descriptions, radiation exposure factors, technical factors, patient age, accession numbers, acquisition numbers, and series descriptions. Additionally, radiation dose values specific to flat‐field imaging and tomosynthesis were separated for analysis.

To maintain confidentiality, patient data was anonymised by remapping internal hospital identifiers to new, anonymised IDs. The mapping history was securely stored within the institution's document storage to safeguard the integrity of anonymisation.

### 2.1 Imaging Protocol

Participants who underwent imaging at Site 1 were scanned using a Hologic Selenia Dimensions system (Hologic, Massachusetts, USA), while those imaged at Site 2 were scanned using a Philips MammoDiagnost system (Philips, Amsterdam, The Netherlands). Only the system at Site 1 was able to perform DBT. Both sites performed imaging using the same hospital protocols for craniocaudal (CC) and mediolateral oblique (MLO) views of each breast, with supplementary views acquired as clinically indicated. Although both vendors support automatic exposure control (AEC), technologists occasionally overrode AEC at their discretion, consistent with departmental practice. A 0.3 mm focal spot size was used as the standard, with focal spot sizes of 0.1 and 0.15 mm used once each.

### 2.2 Radiation Dose Analysis

Entrance surface air kerma (ESAK) in mGy is preferred for comparing radiation doses across mammography models and studies, with ESAK automatically calculated upon each acquisition. Glandular dose, in mGy, is also used within the analysis.

The imaging systems at each site both use the Dance et al. [11] methods to measure glandular dose, assuming a 50:50 granularity assumption. Both systems have been assessed for differences between displayed and calculated mean glandular dose across a range of kVp. As per the ACPSEM, displayed and recorded AGD values should align with Dance model estimates within a 25% variance threshold (ACPSEM, 2022, p.150) [12], with site testing history confirming all records are within a 20% variance from standard.

### 2.3 Statistical Analysis

Significance testing was performed at a 95% confidence interval to ensure that observed differences and relationships were likely to be real rather than due to random chance. The tests used were Shapiro–Wilk, Mann–Whitney *U*, and Pearson correlation. Normality was assessed using Shapiro–Wilk testing and *q*–*q* plots; independence of observations was assumed for the Mann–Whitney *U* test, and linearity was considered for Pearson correlation.

Shapiro–Wilk testing (*p* < 0.001) and *q*–*q* plot diagonal line deviations confirmed that both modality datasets were non‐normally distributed. Consequently, non–parametric Mann–Whitney *U* testing was used to compare dose distributions between groups, as it assesses central tendency without assuming normality. Pearson's correlation coefficient was applied to examine linear relationships between continuous variables, such as patient part thickness and ESAK, after confirming approximate linearity and non‐heteroscedasticity. These analyses were conducted using IBM SPSS Statistics 29 (IBM, New York, USA) and Microsoft Excel (Microsoft, Washington, USA). Limitations of these methods include the small sample size affecting generalisability, the Mann–Whitney *U* test comparing distributions rather than means, and Pearson's correlation being sensitive to outliers and assessing linear relationships only.

Calculations for significant differences between Sites 1 and 2 FFDM ESAK data were also performed to minimise potential bias from variations in imaging systems. However, due to the much smaller FFDM sample size at Site 1 (*n* = 15) compared to Site 2 (*n* = 164), differences could not be reliably assessed, and statistical comparison was not conducted. Similarly, DBT ESAK was not considered for site comparisons, as Site 2 did not provide DBT imaging. Patients who underwent multiple procedures were treated as independent cases, with no data weighting applied, which potentially introduces bias. Descriptive statistics were used to summarise patient factors (age and breast thickness) and technical factors (kVp, mAs, exposure time, and dose).

## 3 Results

### 3.1 Statistical Testing

**FIGURE 1:**
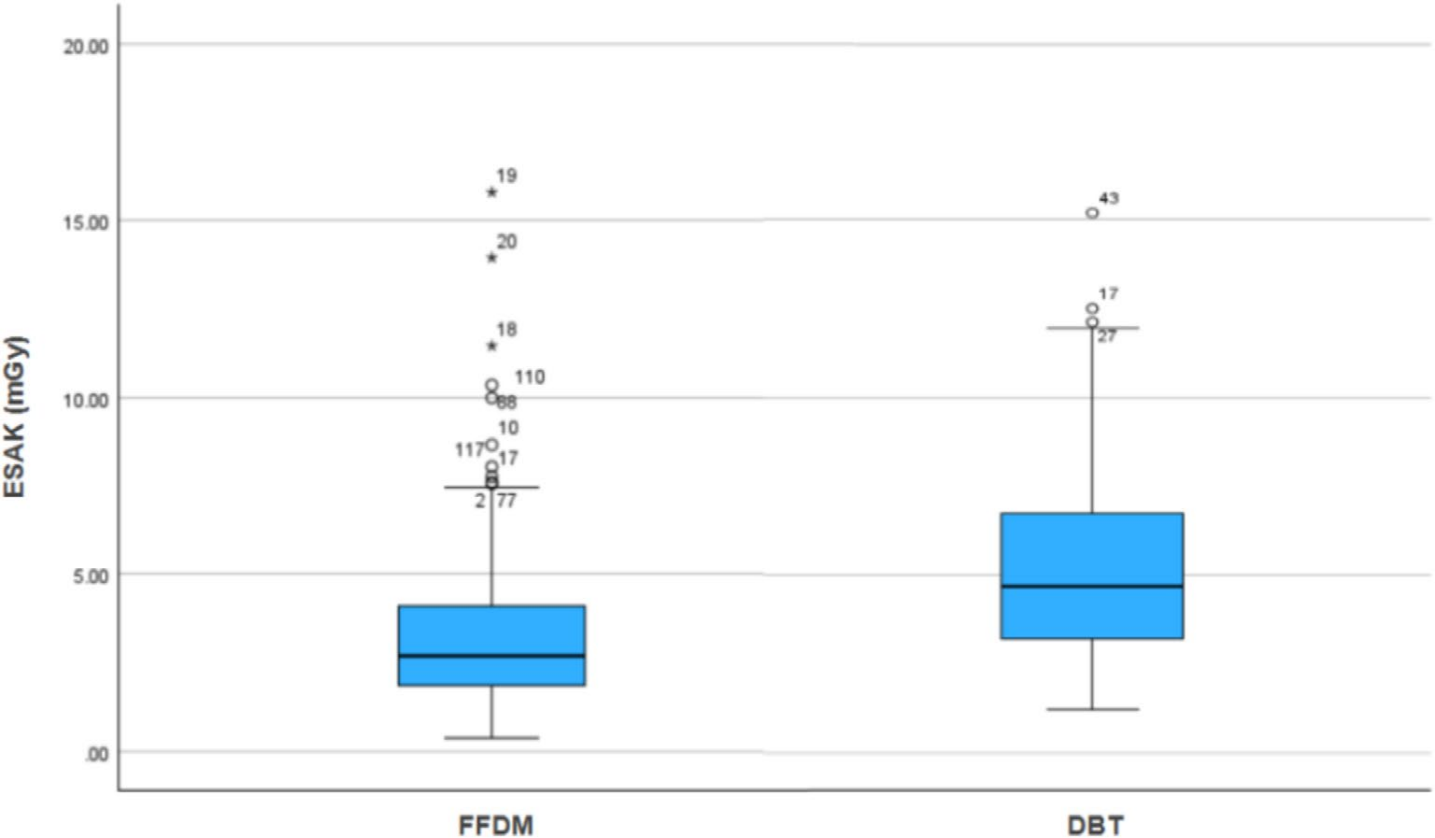
Box and Whisker plot of DBT and FFDM ESAK dose data.

Dose values were recorded and visualised, with Figure 1 below visualising the spread of ESAK dose values across FFDM and DBT. The whiskers indicate the range of dose values, the bolded line the median and the blue area the interquartile range. Extreme outliers are represented with an asterisk, and mild outliers with a circle. Calculations of the FFDM and DBT ESAK doses were performed to determine if there was a significant difference between the doses for both modalities. Shapiro–Wilk testing for both modality datasets revealed a *p* < 0.001, concluding non‐normal distribution and resultant Mann–Whitney *U* testing requirement. Similarly, both *q*–*q* plots contained deviations from the diagonal line, further suggesting a non‐normal distribution.

A Mann–Whitney *U* test was performed to evaluate whether the ESAK dose differed by modality use of FFDM or DBT. The results indicated that DBT had a significantly greater dose than FFDM, *z* = [−6.13], *p* < 0.0001.

### 3.2 Radiation Dose

Descriptive statistics of relevant variables across all examinations are visualized below in Tables 2, 3, 4, 5.

**TABLE 2:** Descriptive statistics for Site 1 patient and exposure variables.

	Site 1				Site 2				Sites 1 and 2 combined			
	Mean	Median (1st–3rd Quartile)	IQR	Range	Mean	Median (1st–3rd Quartile)	IQR	Range	Mean	Median (1st–3rd Quartile)	IQR	Range
Age (years)	67.5	65	2	27 (63–90)	63.91	66	15	61 (26–87)	64	65	15	64 (26–90)
Breast thickness (mm)	56.9	56	9	28 (44–72)	49.23	48	16	69 (16–85)	51	51	19	69 (15–86)
FFDM KvP	29.8	30	2	4 (28–32)	27.6	27	3	10 (24–34)	27.8	28	3	10 (24–34)
FFDM mAs	115.3	110	30	60 (100–160)	125.74	131	56	169 (20–189)	124.9	128	54	169 (20–189)
FFDM exposure time (ms)	1014.1	1128	288	603 (592–1195)	631.41	569	24.5	1299 (507–1806)	663.5	570	74	1299 (507–1806)
FFDM ESAK (mGy)	5.61	5.74	2.76	6.57 (2.09–8.66)	3.20	2.65	2.16	15.43 (0.37–15.8)	3.4	2.7	2.3	15.43 (0.37–15.8)
DBT kVp	29.4	29	3	8 (25–33)					29.4	29	3	8 (25–33)
DBT mAs	107.6	100	10	140 (50–190)					107.6	100	10	140 (50–190)
DBT exposure time (ms)	965.5	975	364	1327 (400–1728)					965.5	975	364	1327 (400–1728)
DBT ESAK (mGy)	5.37	4.7	3.54	13.98 (1.2–15.18)					5.37	4.7	3.54	13.98 (1.2–15.2)
FFDM glandular dose (mGy)	0.896	0.800	0.41	2.95 (0.16–3.11)	1.461	1.53	0.53	1.46 (0.66–2.12)	0.944	0.83	0.44	2.95 (0.16–3.11)
DBT glandular dose (mGy)	1.405	1.34	0.713	2.89 (0.52–3.41)					1.405	1.34	0.713	2.89 (0.52–3.41)

**FIGURE 2:**
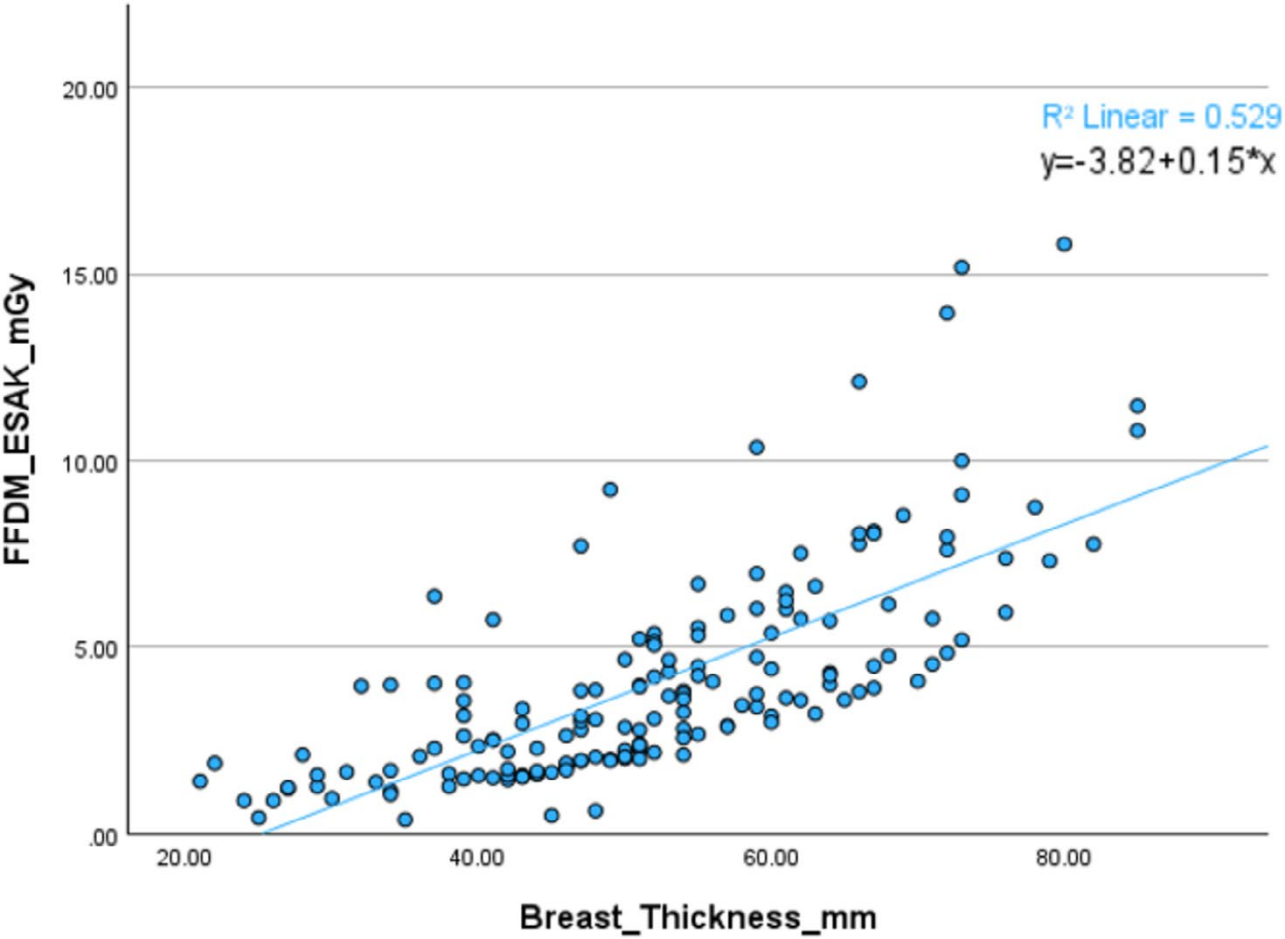
Scatter plot visualising correlation between patient part thickness (mm) and FFDM ESAK (mGy).

The relationship between patient part thickness and ESAK was also plotted, as shown in Figure 2. There is a strong, positive relationship between part thickness and ESAK, *r* = 0.72, *p* < 0.001.

## 4 Discussion

At a median ESAK of 4.7 mGy and glandular dose of 1.34 mGy, DBT delivered a higher dose per image than FFDM, which recorded a median ESAK of 2.7 mGy and AGD of 0.83 mGy. This finding consolidates current knowledge that DBT generally imparts a higher radiation dose due to multiple acquisitions and longer exposure times, supported here by DBT's average 965.5 ms compared to FFDM's 663.5 ms. DBT's higher average dose validates FFDM's use as the primary modality, particularly for patients requiring repeated imaging. Beyond this analysis, mean values have been used for ease of comparison to previous literature.

More notably, FFDM doses in this male cohort were lower than those reported for female cohorts in previous literature and lower than those reported in the Sulieman study [10], a male cohort study. This is likely attributed to differences in anatomical structure, where men have less glandular, adipose, and fibrous tissue, resulting in a reduced average breast density and required radiation dose. Consequently, adequate image quality is achieved at lower doses, even with comparable kVp and mAs values to female imaging, as shown in Table 3 with this study's lowest ESAK and AGD to all compared female cohorts. This anatomical influence is supported by the lower average participant breast thickness in this study (51.3 mm) compared to Sulieman et al.'s male cohort (56.7 mm), and by shorter average exposure times (663 vs. 796 ms). These differences suggest that male‐specific protocol optimisation could further reduce dose while maintaining diagnostic quality.

**TABLE 3:** Comparison of found FFDM dose results to previously published studies.

	This study (mean)	Sulieman et al. (2022) [10]	Tamam et al. (2021)^a^ [13]	Sulieman et al. (2019)^a^ [14]	Baek et al. (2017)^a^ [9]	Tahiri et al. (2021)^a^ [15]	Lekatou et al. (2019)^a^ [16]	Tsapaki et al. (2008)^a^ [17]
Imaging purpose	Both	n/a	Screening	Diagnostic	Screening	Screening	Screening	Screening
FFDM kVp	27.8	28.64	29.1	29.6	28.1	30.96	29.9	28.1
FFDM mAs	124.9	131.2	115.7	81.9	89.6	53.43	106.1	67
ESAK (mGy)	3.4	5.3	5.19	n/a	n/a	6.96	8.98	5

^a^ Indicating female cohorts.

**TABLE 4:** Comparison of patient and exposure variables and dose for FFDM between this study and Sulieman et al. 2022.

	This study				Sulieman et al. 2022 [10]	
	Average	Median	IQR	Range	Average	Range
Age (years)	64	65	15	64 (26–90)	45	57 (23–80)
Breast thickness (mm)	51	51	191	69 (16–85)	56.74	127 (18–145)
FFDM kVp	27.79	28	3	10 (24–34)	28.64	11 (24–35)
FFDM mAs	124.87	128	54	169 (20–189)	149	147 (28–175)
FFDM exposure time (ms)	663.48	570	74	1299 (507–1806)	795.82	3031 (141–3172)
FFDM ESAK (mGy)	3.4	2.7	2.27	15.43 (0.37–15.8)	5.3	27.03 (0.47–27.5)
Glandular dose (mGy)	0.944	0.83	0.44	2.95 (0.16–3.11)	1.47	n/a

**TABLE 5:** Comparison of found glandular dose results to previously published studies.

	This study (mean)	Sulieman et al. (2022) [10]	Tamam et al. (2021)^a^ [13]	Tahiri et al. (2021)^a^ [15]	Young & Oduko (2016)^a^ [18]	Zira, Nzotta (2018)^a^ [19]	Joseph et al. (2018)^a^ [20]	Baek et al. (2017)^a^ [9]
FFDM AGD (mGy)	0.944	1.47	1.3	1.34	2.5	1.3	1.04	1.81
DBT AGD (mGy)	1.405	n/a	n/a	n/a	n/a	n/a	n/a	n/a

^a^ Indicating female cohorts.

In male mammography, acquisitions are almost exclusively performed for diagnostic purposes rather than population screening, typically in the context of symptomatic presentation or follow‐up for known pathology. Table 3 outlines the imaging purposes for this and compares previous literature. The need for specialised views in diagnostic mammography typically incurs an increased exam dose. Whilst this study reports acquisition, not exam dose, the diagnostic context remains important as specialised views entail variable positioning and tailored exposure settings. These factors influence dose values, which differ from the standardised two‐view protocols in female screening cohorts. Recognising these differences in clinical pathways is essential when interpreting dose comparisons between sexes and reflects the unique diagnostic nature of male imaging and anatomy.

An area of potential bias within this study involves the inclusion of 16 manually set exposure factors in the dataset and the use of different AEC algorithms per system. Inconsistent exposure selection limits comparison of dose across examinations and other studies; however, these manually set exposures were kept to maintain the data set size and acknowledge valid clinical reasoning for manual exposure selection, such as the inability to position male breast tissue over the AEC chambers, and cited studies from Saudi Arabia, Nigeria, South Korea, and the United Kingdom each have different demographic, anatomical, and imaging practice variations. This incurs limitations with comparability of statistics such as breast composition, imaging protocols, and radiation doses from studies across different geographic regions.

Limitations to the method of this study include the small sample size, dose analysis not considering the diagnostic quality of acquired images, largely varying sample sizes between sites, the use of two different imaging system manufacturers, and multi‐study individuals over‐representing patient factors. These limit the generalisability of findings to the broader male population.

Recommendations for future research to limit bias include conducting multicentre studies to pool data from different institutions and machines to increase the sample size and generalisability of the results. This more consistent distribution of procedures across the sample would more likely enable the use of parametric tests, enhancing comparability. Data weighting could be implemented by assigning lower weights to patients with multiple procedures, helping to account for the skew in participant factors caused by patients undergoing multiple procedures. Future studies could focus on a cohort of female patients undergoing diagnostic mammography within the same tertiary centre and during the same period as this study. This approach would help eliminate confounding variables and provide more unbiased data, potentially valuable for establishing male diagnostic reference levels that could provide a benchmark for dose optimisation and support consistent practice across institutions.

## 5 Conclusion

This study provides the first Australian male mammography data on ESAK and glandular dose per acquisition, which can serve as a reference for comparison or future protocol optimisation. The findings confirm that DBT generally delivers more radiation than FFDM, highlighting the importance of considering dose implications as its use in breast imaging expands. The lower radiation observed in male FFDM compared to female cohorts suggests that anatomical differences between sexes likely influence mammography doses, supporting the potential for male‐specific protocol optimisation. Future research should combine data from multiple centres, account for patients with multiple procedures, and compare male and female cohorts scanned at the same sites and period to provide clearer insight into gender‐related differences in radiation dose and guide safer imaging practices.

## Author Contributions

Mohamed K. Badawy, Maeve Masterson, and Daniel Carrion contributed to the study's conception, design, and data collection. Zechary Ng performed material preparation, data analysis, and manuscript drafting under the guidance of Mohamed K. Badawy, Maeve Masterson, and Daniel Carrion. All authors commented on previous versions and read and approved the final manuscript.

## Ethics Statement

As a retrospective, observational study, the author's Institute Research Ethics Committee has confirmed that no ethical approval is required. This project was granted an exemption from full ethics review by the institutional ethics board as a quality improvement activity. HREC reference number: QA/91812/MonH‐2022‐343316(v1) and Local HREC reference no. RES‐22‐0000‐740Q–91812.

## Conflicts of Interest

The authors declare no conflicts of interest.

## Data Availability

The data that support the findings of this study are available from the corresponding author upon reasonable request.
